# Evaluating Success and Challenges of a Primary Care Youth Mental Health Programme Using Complexity, Implementation Science, and Appreciative Inquiry

**DOI:** 10.7759/cureus.58870

**Published:** 2024-04-23

**Authors:** Anthony Dowell, Maria Stubbe, Abigail Dunlop, Dasha Fedchuck, Tracey Gardiner, Sue Garrett, Sarah Gordon, Jo Hilder, Fiona Mathieson, Rachel Tester

**Affiliations:** 1 Primary Health Care and General Practice, University of Otago, Wellington, NZL; 2 Psychological Medicine, University of Otago, Wellington, NZL

**Keywords:** co-design, systems science, young people, scale up, primary care mental health, case study, appreciative inquiry, implementation science, complexity science

## Abstract

Background

Using an innovative framework of complexity and implementation science, with underpinning core values of appreciative inquiry (CIS-A), this paper describes the evaluation of a pilot service in New Zealand aiming to deliver integrated psychological therapy services within primary care, to young people (aged 18-24) experiencing mild to moderate mental distress.

Method

Using mixed quantitative and qualitative methods and multiple data sources, there was a strong focus on local innovation and co-design with service users, young people and multiple care providers. Data is drawn from service users, stakeholders and providers of the service in three areas of the lower North Island of New Zealand.

Results

The Piki pilot provided a significant and innovative enhancement of mental health care to this population. The service supported 5307 individuals with a range of therapy options, with the majority having between one and three therapy sessions. From 730 service users who completed a survey, 591 (81%) were ‘very satisfied’ with the service provided. The CIS-A framework was used successfully to provide rapid feedback and encourage adaptation to evolving issues. These included unexpected workload pressures, changes to therapy delivery, the integration of co-design and prioritising the needs of vulnerable groups. There was a successful incorporation of youth/service user input to co-design the programme, introduction of a peer-to-peer support service, and integration of a digital resource platform. The framework was also used to address challenges encountered and to support necessary adaptations in response to the COVID-19 pandemic.

Conclusions

We describe the use of an embedded evaluation to support and inform the implementation of a novel and innovative youth mental health programme. Complexity and implementation science, underpinned by the core values of appreciative inquiry (CIS-A), were successfully utilised providing potential learning that can be applied locally, nationally and internationally. This study has a focus on youth mental health but the principles and utility of applying a complexity and implementation science approach have application in many different health care settings. The use of a framework such as CIS-A can support complex innovation and implementation and can be used to enable rapid course correction and turn ‘hindsight to foresight’.

## Introduction

The last decade has seen a strengthening of psychological therapy services in many countries [1]. In New Zealand, an innovative pilot service aimed to deliver improved access to a range of evidence-based psychological therapies, to young people (aged 18-24) experiencing mild to moderate mental distress (Piki) [2]. The design of the evaluation of the pilot incorporated a developed framework of complexity and implementation science, with underpinning core values of appreciative inquiry (CIS-A) [3]. This paper describes how the CIS-A evaluation framework was applied in the context of this pilot and describes its use in supporting success in innovation and providing suggestions for ‘course correction’ in the development of the service. 

Background to the development of the pilot programme

Worldwide, mental-related distress and substance use-related distress are leading causes of morbidity and disability among young people. They are highly prevalent yet, until recently in contrast to the focus on adult mental health, young people have received relatively little attention [4]. The causes of mental distress in young people are both complex and multifactorial.

In New Zealand (as in other OECD countries), primary care is a major contributor to psychological therapy provision and for the last 15 years, a platform of mental health services has been provided through primary health organisations (PHOs) which fund the delivery of those services, either directly or through contracted providers. Recognising an increasing need for mental health services for youth, the Ministry of Health (MoH) released a tender seeking proposals to pilot a service to improve capacity, capability and equity of access to psychological therapies for young adults aged 18-25 years experiencing mental and substance use-related distress.

The programme brief was to develop a model of care based on the UK-based IAPT (Improving Access to Psychological Therapies) service programme using largely standard Beckian-based Cognitive Behavioural Therapy CBT [5], but adapted to fit the New Zealand context, and incorporating a number of specific innovations.

Services were intended to provide support from a strength-based approach with an emphasis on self-management skills. The pilot was to be integrated with general practice, Youth One Stop Shops (specialist youth health services), and tertiary institutions to allow onsite service delivery, immediate booking, self-referral and short waiting times. There was to be a particular focus on service to population groups known to experience the greatest inequities and disparities, (including those from low-income, Māori - indigenous population of NZ, Pacific, migrant and LGBTQIA+ communities).

Specific innovations intended to integrate with and enhance the provision of psychological therapy, included the establishment of mechanisms for co-production to inform the design, delivery, implementation and evaluation components of the programme; the provision of structured peer support services (individual or group-based) where support is provided by people with experiences of similar types of distress; a self-referral website with resources; and a digital app featuring online forums, ability to message a “coach”, resources and a diary function. The programme also included an already established service providing phone counselling. There was a strong focus on attempting to brand the programme in a way that specific vulnerable groups would be receptive to it. The initiative was named ‘Piki ‘, a Māori word meaning both ‘to support or aid’, and ‘to climb or ascend’, and was available to young people in the Greater Wellington region of New Zealand.

This paper explores the use of the CIS-A framework as an evaluation method and tool to determine facilitating factors and challenges encountered in the development and implementation of this pilot. 

## Materials and methods

The evaluation framework

The evaluation framework combined well-established methodologies for advancing quality in health services delivery, including the use of mixed quantitative and qualitative research paradigms. Much of the framework was based on earlier evaluation experience of the Ministry of Health-funded Primary Mental Health Initiatives (PMHIs), which was conducted between June 2005 and November 2007 [6]. The main objectives of the Piki evaluation were to assess how well the pilot achieved its stated objectives, and to what extent innovations could be successfully introduced and potentially used in a possible future national rollout of the initiative.

In recognition of the complex nature of the project, the evaluation was designed from the outset to be an embedded part of the pilot programme: findings were to be integrated iteratively into the development and delivery of the pilot service by reporting in real time on progress to the service delivery organisations, the New Zealand Ministry of Health and Government.

The evaluation itself incorporated also co-production with an academic service user team and a group of youth service users who collaborated with the other researchers on all aspects of the evaluation process including the development and agreement of outcome measures, service user participation in interviews and other research processes (e.g. construction of interview schedules, analysis of data and interpretation of results).

**Figure 1:**
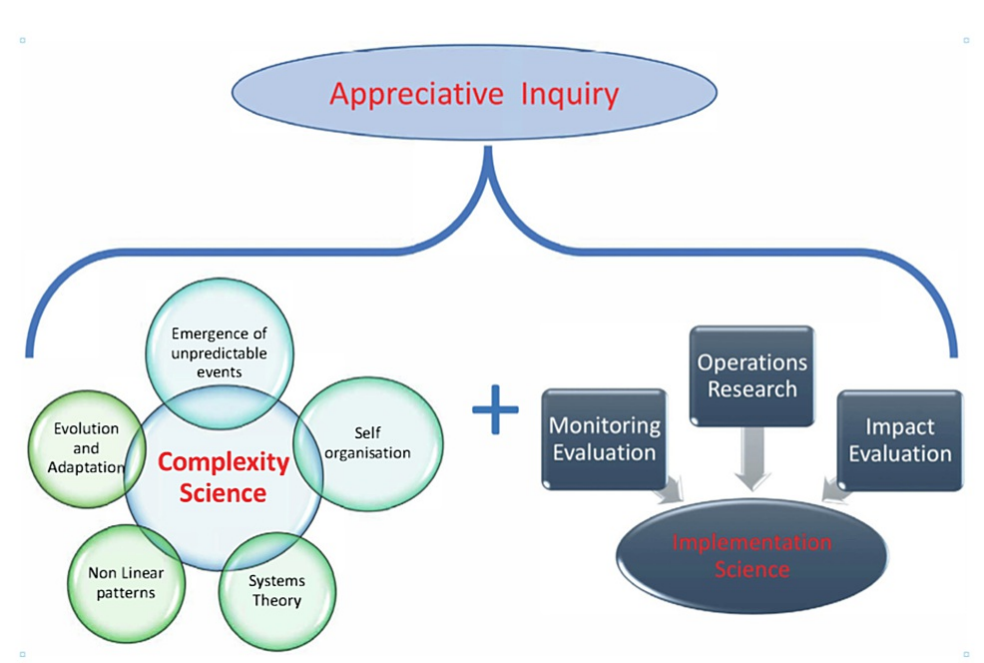
A framework of complexity and implementation science using appreciative inquiry (CIS-A) Image Credits: Anthony Dowell

Figure 1 shows the overarching framework used for the evaluation; this is based on the principles of complexity and implementation science, with an underpinning platform of ‘appreciative inquiry’ [7] which seeks to elicit a strengths-based approach to evaluation activity and analysis from the standpoint of a ‘critical friend’. An appreciative inquiry approach focuses on the positive attributes inherent within existing systems retaining and building on the facilitators or best elements of current practice and seeks to engage stakeholders in a process of self-determined change.

This particular framework had initially been developed for exploring complexity and implementation challenges in immunisation delivery [3]. The overall aim of the approach is to support local sustainable innovations and knowledge transfer, built in from the beginning of a programme

Within this health service context, complexity science acknowledges the need to explore organisational and systems change from multiple perspectives, utilise non-linear approaches and recognise that often ‘predictably unpredictable’ consequences emerge during interventions. It also acknowledges challenges as learning opportunities [8]. Implementation science promotes the systematic uptake of research findings and other evidence into routine practice and also offers insights to enhance the performance of health sector delivery [9].

**Figure 2:**
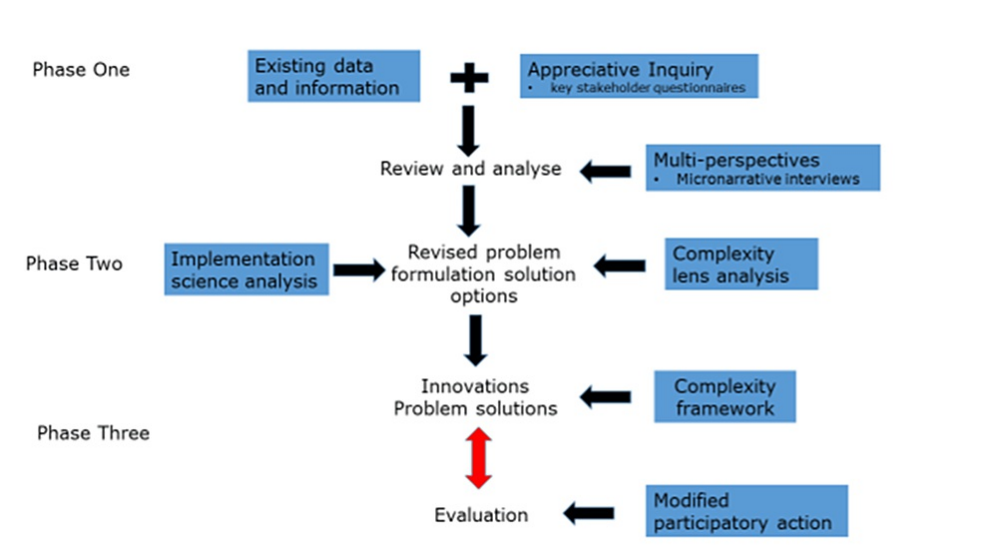
Process and phases in using the CIS-A framework. Image Credits: Anthony Dowell

The generic approach to the operationalising of the framework is shown in Figure 2. Activity begins with an appreciative inquiry into any existing available data that complements purposively collected local narratives and key stakeholder interviews. Analysis uses established complexity science approaches and tools such as sense-making [10]. Implementation methodologies include Donabedian's framework for Health Services Evaluation to sequence the evaluation activities [11] and participatory reflection-action approaches adapted to account for the overall strategic direction built into individual projects [12]. Evaluation is core and involves an ongoing iterative process.

This paper explores the use of the CIS-A framework as an evaluation method and tool to determine facilitating factors and challenges encountered in the development and implementation of this pilot. 

Setting and participants

Services available through the pilot were psychological therapy based in three Primary Health Organisations, and two Tertiary Education Counseling Services, a Peer Support service, a telephone counselling service and a service providing more intensive therapy via clinical psychologists. Services were provided to any young person between the ages of 18 and 25, living in the greater Wellington region and were accessible via self-referral, GP/practice nurse referral or via support organisation referral. 

Quantitative methods

The quantitative component of the evaluation involved ongoing collation and analysis of service utilisation and outcome measurement data routinely collected by the primary health organisation and other providers and analysed using a cohort design to analyse individual service user data.

Data collected included service utilisation (e.g. demographics, types of services accessed, duration of engagement), workforce data, and outcome data (in the form of psychological measures such as PHQ-9, WHOqol and GAD-7) from the prospective cohort of service users. Online surveys were administered to therapists, peer supporters and service users at different time points during the evaluation. Service users were also asked for feedback on therapy contact session effectiveness through the Session Rating Scale (SRS), and Outcome Rating Scale (ORS) [13]. Data from Cognitive Behavioral Therapy (CBT) training, including CBT assessment results and course feedback, were also collated.

The original intention was to collect psychological measures at several points in the Piki client journey, with all clients offered the opportunity to complete all measures. In response to strong feedback from the service users group and wider discussion with partner organisations, it was decided that completing outcome measures would not be compulsory. There was debate about which measures were most appropriate. Completion rates were not high: baseline PHQ-9 and GAD-7 measures (intended as screening tools) had a 42% completion rate; WHOQOL-bref (a quality-of-life measure) had 11% completion; Session Rating Scales (SRS) had 10% completion; Outcome Rating Scales (ORS) had 20% completion. Factors affecting (lack of) completion included the choice of measures, practical/workload considerations, and the impact on the therapeutic relationship.

Analysis of surveys of service users and therapists used descriptive statistical counts of question responses. Before and after outcome measures of service users' interactions with therapists and other components of the Piki service were undertaken and recorded as part of usual clinical practice.

Qualitative methods

A range of qualitative methods was used, including (i) iterative cycles of individual, small group and focus group interviews of key informants, service users and service providers, (ii) participant observation and structured reflection throughout all stages of the pilot by evaluation team representatives on all programme steering, advisory and operational groups, and (iii) review and coding of all key programme documents.

Evaluation team members attended and undertook participant observation of, and critical reflection on, all steering, advisory, operations and reference group meetings throughout the pilot in addition to key workshops and public events. These observations were supplemented by a review of correspondence, official minutes and other planning documents, media and communications material, and input from the youth and service user reference groups, to inform a robust analysis of process themes.

Interviews and focus groups were conducted with 119 individuals in total from three categories: those we have called ‘key informants’ who included those involved at the design and management level and members of the governance and operational groups; service providers (therapists/counsellors, peer supporters); and service users.

All interviews and focus groups were transcribed by an independent transcriber. These transcripts, and all documents collated and created as part of the process evaluation, were imported into an NVivo file and coded using a content framework as well as more inductive thematic codes that were developed by the evaluation team. In addition to content coding, relevant sections were coded for ‘‘sentiment’’ i.e., as either positive or negative.

A separate additional process was followed for the analysis of the service user interviews. The analysis of this data was conducted primarily by the researcher who interviewed the participants but also included direct involvement from the Service User Reference Group.

Integration of quantitative and qualitative findings

From the outset, integration of the various data sources and analyses described above was a key focus and has been modelled on evaluation methods used in projects where complexity, implementation and 'scale up' have been identified as important factors in the overall evaluation assessment [3,6,9,14]. The full evaluation team participated in regular meetings (weekly or fortnightly depending on the project stage) where emerging findings from different parts of the evaluation were reported, discussed and critiqued. Integrated summaries were also fed back to the service providers, youth reference group, programme steering, advisory and operational groups and the Ministry of Health via interim and final reports with a focus on specific emerging issues identified in the course of the evaluation (e.g. targeting of priority groups, transition to telehealth during the COVID-19 lockdown, therapist surveys).

Ethics committee approval

Consent was obtained or waived by all participants in this study. The University of Otago ethics committee issued approval H19/044 for this study.

## Results

Quantitative summary

The following summary of quantitative Piki activity provides context for the integrated mixed methods analysis using the CIS-A framework. Overall 5307 individuals accessed the Piki service in the two years up until the end of December 2020 (2090 in 2019; 3217 in 2020). Of those, 3465 (65.3%) were female and 3710 (69.9%) were European. Māori comprised 727 (13.7%) of clients, while Pacific youth comprised 186 (3.5%); these two priority groups thus accessed the service at levels below their population share (17.4% and 8.0% respectively based on 2018 census data). The majority of Piki clients had between one and three sessions, with 1751 (33%) having only one session. A substantial minority of service users numbering 244 (4.6%), had 13 or more sessions.

From 730 service users who completed a satisfaction survey, 591 (81%) reported feeling very satisfied or satisfied with the number of therapy sessions they received and 547 (75%) would recommend Piki to a friend.

Integrated themes

The following themes were identified in which elements of CIS-A were used in the exploration of emergent problems or implementation pathways.

Complexity in Governance and Advisory Group Organisation

The governance of the project was complex, particularly in terms of incorporating innovation aspects such as co-design. Three committees of predominantly service provider and organisational stakeholders were established: a project steering group, meeting monthly; a clinical advisory group, meeting monthly; and a project operational group, meeting weekly or fortnightly.

The composition of these groups evolved over the course of the project, with attempts to ensure appropriate representation of youth, service users and cultural groups along with the appropriate service providers and stakeholders.

Input from youth and service users was sought via two routes: a Service User Reference Group (SURG) and a Piki Youth Reference Group (PYRG). The SURG was created to support the co-design process, with a specific focus on evaluation. Its members are youth with lived experience of mental- and substance use distress and experience of primary care services as a result. Eleven people aged 17-24 were initially recruited to the SURG with intentional recruitment of young people identifying as indigenous Māori, disabled, and Rainbow. The group was facilitated by a service user academic on the evaluation team. The group co-designed interview and focus group schedules discussed the acceptability of quantitative measures, provided feedback on peer support processes, and assisted in the analysis of interview data.

The PYRG was created to represent the youth population more generally. This group provided advice in terms of youth culture, environment, communication, accessibility of services and marketing. It helped to co-produce communication strategies and design of the website and logo as well as considering and advising on outcome measurement and therapy options. The group comprised approximately 19 members including members identifying as Māori, Pacific and from the Rainbow community.

Appreciative inquiry approaches were used on a number of occasions to review and adjust group membership and operations, with support from the evaluation team.

Time Frame and Implementation Expectations

The initial proposal was for a three-year project, including a six-month service development phase before the commencement of new service delivery. Following the awarding of the contract, the Ministry of Health reduced the time frame to 2.5 years. This reduced timeframe introduced elements of complexity in terms of the need for expectation management and a significant amount of reframing of implementation pathways. Following an impact evaluation assessment, changes were made to the original project plan including a reduction in the target number of young people receiving services from an estimated >10,000 over 3 years to >8,000 over two years and a conflation of the design and service roll-out phases.

There was also close monitoring of the integrated services that comprise the Piki model. Each of the new components was phased in over a number of months with the suite of support options introduced successively as they were developed.

The compressed project timeframe for Piki necessitated both an unusually rapid development of branding and a progressive rollout of social marketing and engagement activities as each new service component or provider became live rather than a single official launch preceded by comprehensive consultation.

Appreciative inquiry and the evidence base created by the evaluation of the previous platform of primary mental health services. was used to manage expectations regarding the implementation of the pilot from the previous platform of existing services [6].

The evaluation team also recognised that the change in timeframe reduced opportunities for co-design of the evaluation methodology. An example was the need to adapt anticipated clinical assessment outcome measures when concern was raised by the service user and youth reference groups about their utility and cultural (youth and ethnicity) appropriateness.

Having the evaluation embedded in all programme workstreams from the start meant that despite this time ‘compression’ we were afforded a unique opportunity to observe the unfolding complex implementation processes and provide immediate observational feedback and appreciative/critical input.

Patient Engagement Pathways

From the project's outset, there was inherent complexity in the multiple ways it was intended that service users would access care. These numerous referral mechanisms were designed to provide service users with a number of options to “get to” the service and included self-referral through the Piki website, and referral from multiple provider groups and types including primary care, Youth health services, and social support organisations (Figure 2). The intention was for all referral pathways to lead to an initial face-to-face therapist session during which assessment and registration with Piki are conducted.

Due to a number of factors in the first six months of operation, there was unexpected pressure on the referral process, and complexity theory was utilised to provide a number of options to manage the tension between open access to the service of choice and maintaining a reasonable wait time.

An example of an unexpected emergent event linked to branding and marketing is described: The Pilot service was officially launched at a local Māori Marae (meeting house) in February 2019, followed by a second launch at a local university in May 2019. Following the university and the website launch, there was a spike in self-referrals, with over 80 in the first week, but this subsequently settled to 17-25 per week. The cause of the ‘spike’ was in part due to unrealistic expectations of the capacity of the pilot due to some media communications implying that there would be virtually unlimited and immediate access to the service. This example of a ‘predictably unpredictable‘ event emerging in the complex operating system was addressed following a review by the PHO with an emphasis on appreciative inquiry and considering multiple alternative options to manage the demand.

An intake coordinator role was also established to act as a first point of contact in an attempt to ensure efficient service delivery and a safe ‘triage’.

Training

The training programme was based on well-established and evidence-based content and training methods and on significant past experience from the training team. It was recognised that the transition from an existing platform of delivery to an enhanced programme could potentially lead to a gap between therapists being trained and the expectations of different service providers. An example of the potential gap was that 20% of the therapy workforce did not have prior CBT training.

To attempt to minimise this gap, existing CBT-trained staff attended a two-day fidelity training and adherence to CBT was formally assessed. An additional 23 therapists were recruited and trained, over a year-long course receiving a postgraduate certificate in CBT.

To respond to the particular needs of specific communities, additional competency training was provided progressively for work with Māori, Pasifika and Rainbow service users. There was also sexual harm training, and in-person therapists and peer supporters also received some training in telehealth to prepare them for the sudden need for widespread use of this during the COVID-19 lockdown.

Regular supervision for therapists was part of the Piki model. Supervision for therapists who completed the CBT training with Otago University was provided by psychologists from an external organisation.

Digital Platform

The digital platform consisted of an App and a website. The App was developed from an existing Wellness App and was originally intended to function as a data collection tool for all client referrals. The App included direct access to the clinical assessment and outcome measures as well as psychological therapy session rating measures. It also provided resources, including tracking tools, a mood diary, an online community, and a facility for therapists and clients to message each other.

A number of specific issues were identified during the development and implementation phases of the App. These included a need for after-hours monitoring to manage risk of more serious presentation, lack of clarity over whether the App was an ‘intervention’, how digital engagement fits into the mix of support options and a relatively low uptake by service users.

A website for the project was developed, incorporating input from the PYRG, enabling self-referral (and the option to refer a friend) and providing links to a range of resources.

Equity

Due to concerns about ongoing accessibility barriers experienced by young indigenous Māori and Pacific populations and young people not registered with a primary care provider, the pilot aimed to reach these groups by focusing on them in the initial launch, and in website marketing. There was also the promotion of Māori and Pacific representatives in working and policy groups and awareness of the need to increase the representation of Māori and Pacific practitioners in the workforce; both of these priorities contained challenges given the availability of practitioners and representation.

A further challenge to prioritising equity was observed in the high volume of students as initial service users with associated potentially higher therapist allocation to that group. This risked ‘favouring’ client groups who had traditionally had greater access to services, or at least, creating a perception that this was the case. The evaluation team identified this issue early in project implementation, as did the PHOs. When workload pressures eased in the latter part of the project specific implementation strategies were employed, including community consultation and appointment of staff with ethnicity matched to that of local communities.

Development of a Peer Support Programme

Early in project development, a peer support organisation worked with the two youth groups, and with Māori and Pasifika groups, to co-design a tailored peer support service. 

Overall this innovation was highly regarded by both service users and provider organisations, with the tailored approach from peers with a similar mental health experience being of particular value. The use of the peer support programme also led to debate about where peer support should be positioned within care pathways [15]; the ability of peer supporters to engage in all stages and levels of acuity was perceived positively by service users. Implementation challenges included developing procedures to respond to service user self-harm or suicide ideation or plans and clarification about whether peer support was an option that could be taken up without accessing other elements of the service.

Therapy Content

While initial therapy content was based on the standard Beckian-based CBT as used in the UK IAPT programme, there was a shift by some therapists in the style of therapy delivered. Causal factors included workload system pressures, differing attitudes and commitment to the CBT model by senior management and team leaders in some organisations and therapists wanting to use a wider range of therapy modalities. While therapy was still CBT-informed, variation developed in both the duration of therapy offered and the content of therapeutic sessions. The CIS-A framework was used to explore the advantages and disadvantages of different models of therapy content, with the result of a variety of CBT-informed styles being used and becoming an increased evaluation focus.

Co-design

An important innovation of Piki was the use of co-production in the design, delivery and evaluation of the project.

The CIS-A model was used to explore the tension between the delivery of Piki from a platform of existing services and a set menu of innovation, compared with adopting new directions and priorities identified through the co-design process. Complexity was acknowledged in the compressed timeframe which eliminated a dedicated co-design period. There was some discontent from youth reference groups at times when they felt their input was not being acted on, and when it became clear that fundamental aspects of the model of care had already been decided. Some therapists also expressed discontent with their level of input, as therapists were not included in the co-design processes. There was ongoing discussion throughout the project about problems with communication and understanding of co-design processes, transparency of roles and expectations, and the degree of relevant community involvement for appropriate co-design input. As a result implementation modifications continued throughout the project with contributions from a variety of different sources. 

COVID-19

The COVID-19 pandemic produced very significant challenges for the health sector in New Zealand, as elsewhere. With the announcement of a nationwide ‘lockdown‘ towards the end of March 2020 to prevent community spread, primary care providers made a rapid and unprecedented transition to virtual consultations.

Although conducting individual sessions using telehealth (video or phone) had been a previous option for therapists and peer supporters within Piki, the lockdown necessitated an immediate and complete transition to ‘virtual’ consulting for a limited time. Some clients opted out of telehealth delivery, choosing to wait until usual services resumed, but many did continue with therapy or peer support in this way. The CIS-A framework was useful in the COVID response by emphasising the prior and developing capacity for telehealth, reflecting on self-organisation observed in some provider innovations and highlighting the importance of the emergent literature on negotiating new forms of consultation in primary care settings.

Planned developments such as social media marketing to Māori and Pacific and the creation of a multi-disciplinary team approach to supporting referrals of those experiencing more ‘severe’ distress were delayed or reduced during the initial lockdown and by increased spikes in referrals and wait times following that.

## Discussion

This paper describes a complex intervention in youth mental health and the use of a framework model incorporating complexity and implementation science principles and appreciative inquiry (CIS-A) to assess how the pilot could achieve stated objectives, and enable a rapid response to challenges encountered during the pilot period.

While the project was designed to be similar in overall design to the UK IAPT project [12], New Zealand had previously developed a successful platform for primary mental health service delivery [6]. An appreciation of the value of this platform led to an implementation strategy aligning ‘business as usual’ work for existing services with the ability to scale up [14] the therapy input to the chosen age group and incorporate a number of service innovations. The complex project structure at times led to challenges that are generally faced by mental health projects with multiple interacting elements and governance requirements, such as delays in implementation timeframes and inter-agency communication [16,17]. 

An important innovative feature of Piki was youth and/or service user engagement in co-development of the development, delivery and evaluation of the project. Tight time constraints have been noted as a co-design barrier in other health sector settings, including youth mental health projects, and this was the same for Piki. While compressed time frames are not unusual in healthcare there is relatively little literature on their impact on implementation pathways Ensuring that projects can accommodate meaningful input from service users is a challenge that requires additional focused attendance to actual application in practice.

The original funded project proposal was intended to conform to a model of therapy delivery with a focus on fidelity to CBT as the treatment modality [5]. The implementation rationale for this was evidence from overseas evaluations that treatment fidelity was an important factor in successful outcomes [18]. Not unexpectedly in this complex operating environment, new practices emerged [9], in response to local demands for a more flexible range of therapy options in keeping with other experiences [19].

Our results align with a relatively small literature recognising the value of incorporating thinking from complexity science into understanding this area of health care. In considering the ‘Headspace’ youth mental health project in Australia, Ellis and colleagues highlighted how recognition of this area of care as a complex adaptive system might have ameliorated some of the described concerns of a relatively top down and ‘one size fits all approach [20]. On a number of occasions as described Piki took a more flexible and co-deign approach enabling complex issues to be managed.

Complexity and implementation science appropriately focus on unexpected emergent events and the importance of adapting to challenges with creative innovation in implementation pathways [9]. The impact of the media on health outcomes is significant [21] and in this initiative, the scope and framing of a media promotion had a significant short-term impact on workload, which was managed by temporarily by putting previous priorities on hold.

COVID-19 provided another complex and emergent event with the need for a rapid transition to virtual delivery of services. Implementation of this change in working practice has been extensively recorded across many areas of health activity [22] including mental health from early in the pandemic [23]. In this initiative, the rapid transition to telehealth consultation enabled services to continue, but in line with other commentary, while there was initial acceptability, questions remained regarding service user choice and the impact on those experiencing health and other inequities and disparities [24]. Clinician preference adds additional complexity, something noted in other studies where it has been questioned if clinician resistance is hindering sustained, widespread telehealth innovation [25].

The CIS-A framework was used to explore the tension between the stated aim of providing easy and timely access for all with the need for an early assessment and ‘triage’, for both workload management and clinical safety reasons. Implementing an appropriate balance between access and triage has been recognised for youth services in a number of different settings [26].

Linked to access and referral pathways are concerns about managing workload, particularly in terms of the marketing of such initiatives and how well the workforce can respond to stated aims of improved access to services for those who are currently underserved. There is significant literature highlighting the challenges of reaching underserved populations, whether in terms of ethnicity in New Zealand [27], Canada and Australia, or achieving equity in terms of access for those on low incomes [28]. While complexity and implementation science approaches can help us explore this issue and highlight evidence-based strategies, addressing equity concerns for those with mental distress remains a key challenge for this initiative.

Following the submission of a final evaluation report to the Ministry of Health [29], the Piki service was extended for a further 18 months, with a specific focus on responding to local access and equity issues. 

Limitations

This paper describes the use of complexity and implementation science approaches within a particular context, and while we believe the findings have value for other mental health and broader health service applications, we note the following limitations for this particular project. There was general agreement that in an ideal world, true co-design would mean all elements of the project design and evaluation would have been present from the beginning. This ideal conflicted with the pragmatic imperatives of the need to build on existing services, efficiency, and inherent constraints in funding models. Many of those involved in the development of Piki accepted there were limitations on the extent of ‘true’ co-design due to these constraints. Another limitation was the degree to which the youth/service user group members could usefully provide input about innovative elements of the service of which they had no direct experience (e.g. peer support, telehealth).

## Conclusions

We describe the use of an embedded evaluation to support and inform the implementation of a novel and innovative youth mental health programme. Complexity and implementation science, underpinned by the core values of appreciative inquiry (CIS-A), were successfully utilised providing potential learning that can be applied locally, nationally and internationally.

We were able to explore implementation pathways from multiple perspectives with a view to supporting innovation and provide a rapid response to evolving issues and encountered challenges. Tensions and challenges in the early stages of the programme were thus expected, accepted and on a number of occasions collaboratively negotiated.

There are a number of areas of potential further research following the results of this study. Firstly, mental health issues particularly for young people are an increasingly important area of primary care work, and evaluation of initiatives is essential. Secondly, while this case study has a focus on youth mental health, the principles and utility of applying a complexity and implementation science approach have applications in many different healthcare settings. The use of a framework such as CIS-A can support complex innovation and implementation and can be used to enable rapid course correction and turn ‘hindsight to foresight’.
